# Amphetamine or skin cream? The impact of the sampling site on the concentration of controlled substances: a case report

**DOI:** 10.1007/s00414-022-02838-6

**Published:** 2022-05-24

**Authors:** L. Gessner, B. Madea, A. Maas, M. Krämer

**Affiliations:** Institute of Forensic Medicine, Department of Forensic Toxicology, University Hospital Bonn, Stiftsplatz 12, 53111 Bonn, Germany

**Keywords:** Controlled substances, Amphetamine, Drugs of abuse, Skin cream

## Abstract

This case report demonstrates the impact of different sampling sites on the quantification of narcotic substances. In 2020, officers secured a syringe containing a light-yellow paste-like substance, for which a drug pre-test indicated a positive result for amphetamine, inducing subsequent analyses of the sample by means of a gaschromatographic-mass spectrometric method (GC–MS) and liquid chromatography–(tandem) mass spectrometry (LC–MS/MS). Depending on the sample location, different results were obtained, with amphetamine not being detected in each sample. Amphetamine was particularly found at the outlet of the syringe, while amphetamine detection on the inside of the syringe at the plunger seal was only possible occasionally and, moreover, in lower concentrations. Based on this and with regard to the comparatively small amphetamine concentrations, contamination of the syringe (especially on the tip of the syringe) was assumed. Hence, the results strengthened the importance of the implication of different sampling sites, when either homogenization of the sample is not feasible or is not performed for reasons of plausibility checks concerning possible contamination of the sample.

## Introduction

Amphetamine and its derivatives rank among the most widely abused substances. Yearly, the European Drug Report by the European Monitoring Centre for Drugs and Drug Addiction (EMCDDA) [1] summarizes, e.g., prevalence, seizures, and usage of drugs for the most common drugs of abuse. The prevalence of drugs is reported by European countries and data are collected by the EMCDDA. According to the EMCDDA, the prevalence of amphetamine use for young adults (aged 15–34) was about 1.4% in 2020. In comparison, a prevalence of roughly 3.0% was reported in 2018 and approximately 2.6% in 2017.

In cases where alleged narcotics are secured by, e.g., the police or custom investigators, macroscopic characteristics give first evidence on the type of substance. Amphetamine mostly appears as amphetamine-sulfate as white to light-yellow, water-soluble, and crystalline powder [2] but is also often sold as amphetamine paste [3]. Besides visual inspection, substance pre-tests are widely used as first evidence on the secured substance type. Thereby, drug wipe tests (e.g., by Securetec Detektions-Systeme AG (Neubiberg, Germany)), as well as substance tests (e.g., by ESA®-Test GmbH (Eisenach, Germany) or NIK® Polytesting System (Jacksonville, Florida, USA)) are used. Those are mostly based on color reactions, e.g., Marquis reactions, which are specific for different substances or substance classes.

Since pre-tests only give unsecured evidence on the potential substance type, further targeted analyses are inevitable. The German Society of Toxicology and Forensic Chemistry (GTFCh) provides guidelines for the handling and measurement of narcotics [4, 5]. Hence, single-sample analyses are sufficient for qualitative substance identification, while homogenization of the complete exhibit or of a proportion of a minimum 30% with subsequent double determination has to be performed for exact quantitative analyses.

The following work addresses the importance of the sampling site and homogenization procedure in investigations of narcotics based on an exhibit analysis conducted in our laboratory.

## Case report

In 2020, the police performed a search of an apartment, where the officers secured a substance-filled plastic syringe wrapped with tinfoil. The content of the syringe constituted of a paste-like substance of white to light yellow color. The accused claimed that the substance was a cortisone-containing ointment. In the police agency, a drug pre-test (NIK® substance test) was implemented, showing a positive test result for amphetamine. Hence, the exhibit was forwarded to the forensic laboratory for substance analytics.

## Materials and methods

The analysis of the sample was performed with two analytical methods. A qualitative screening method was conducted on a LC–MS/MS system. Quantitative measurements were carried out on a GC–MS system.

### Standards and solutions

Amphetamine (100 μg/ml) and amphetamine-d5 (1 mg/ml) were obtained from Cerilliant® (Round Rock, Texas, USA); diazepam-d5 (1 mg/ml in methanol) was purchased from LGC Standards (Wesel, Germany); N-methyl-bis(trifluoroacetamide) (MBTFA) was provided by Macherey–Nagel (Dueren, Germany); n-hexane (LC-grade) and acetonitrile (LC–MS hypergrade) (both Supelco®) as well as formic acid (≥ 99.8%) were obtained from Sigma-Aldrich (Taufkirchen, Germany); 1-chlorobutane (≥ 99.8%) and methanol (≥ 99.9%, for HPLC) were purchased from Honeywell Riedel-de Haën (Schwerte, Germany), sodium hydroxide (NaOH) from PanReac Applichem ITW Reagents (Darmstadt, Germany); ammonium formiate (> 99%) and pH 11 buffer solution (ingredients: boric acid, sodium hydroxide solution, potassium chloride) were purchased from Honeywell Fluka (Schwerte, Germany).

### Sample preparation

The obtained plastic syringe (total volume: 1 ml) contained a paste-like substance to a volume of 0.4 ml. With regard to the positive result for amphetamine in a previously performed pre-test, qualitative substance measurements for the possible detection of amphetamine and amphetamine derivatives were performed at first. For the sample preparation procedure, the guidelines of the GTFCh were considered [5]. Concerning these guidelines, single samples are acceptable for qualitative purposes only. Hence, a first single sample was obtained from the inside of the syringe at the plunger seal.

Qualitative measurements were performed on a liquid chromatography-(tandem) mass spectrometry (LC–MS/MS) system. Therefore, a small sample amount of 17.0 mg was withdrawn from the inside of the syringe at the plunger seal. The sample was suspended in an adequate amount of methanol. Two hundred microliter of the dissolved sample was mixed with 20 μl internal standard solution (diazepam-d5, 1 μg/ml) and 50 μL buffer pH 11 solution, was vortexed, and subsequently extracted using 1 ml 1-chlorobutane. After vortexting (1 min) and centrifugation for 10 min at 9888 × *g*, the organic phase was removed and vaporized in an Eppendorf Concentrator 5301 rotary evaporator (Eppendorf AG, Hamburg, Germany) at 30 °C. The residue was solved in 100 μl solvent-mixture (see Instrumentation) in a composition of 50:50 (v/v), before the sample was injected into the LC–MS/MS system.

**Fig. 1:**
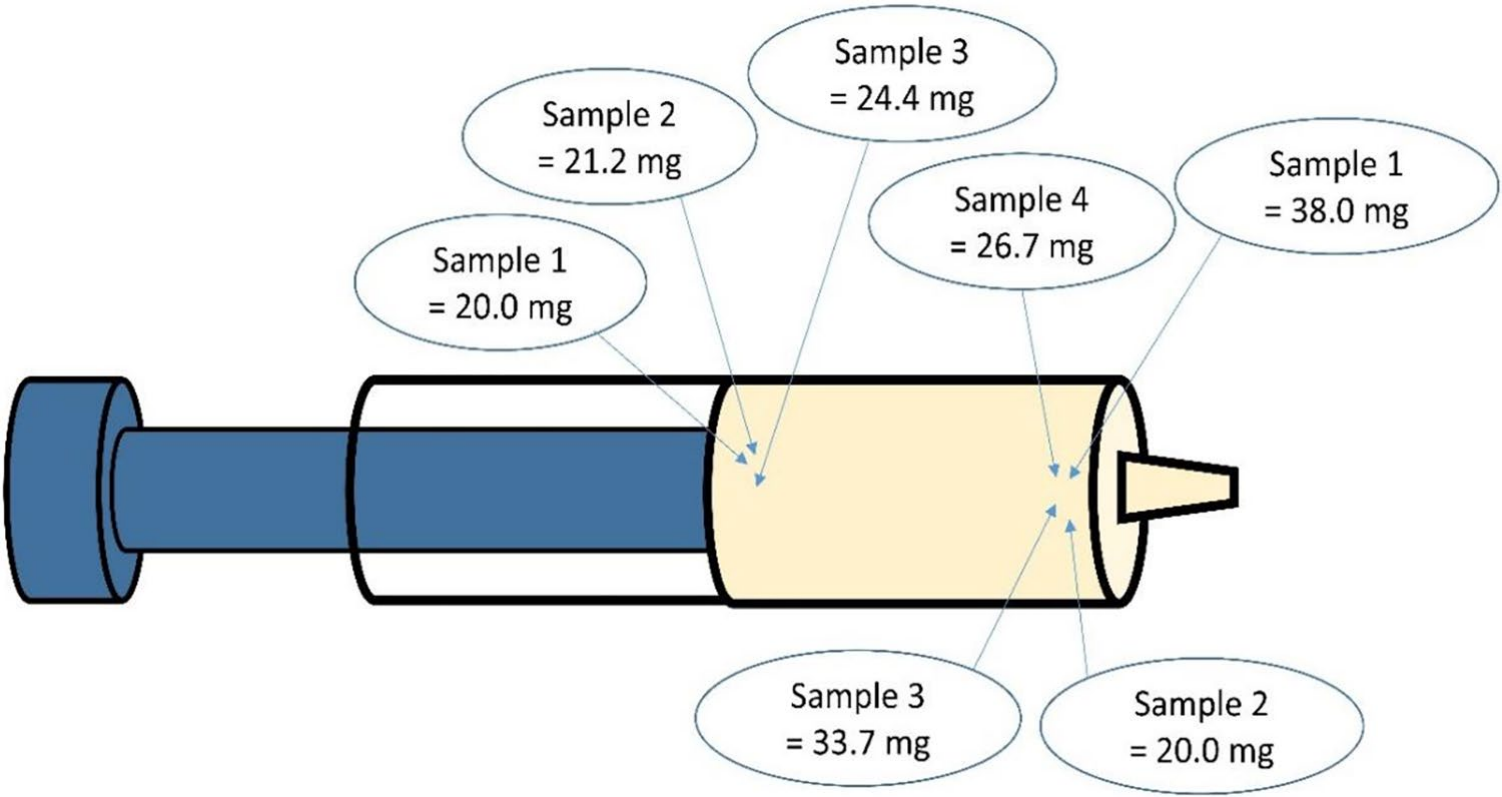
Depiction of sampling locations for quantitative measurements of amphetamine

For quantitative investigations on amphetamine, sample amounts of 20.0–38.0 mg were used. Thereby, three samples were obtained from the inside of the syringe at the plunger seal and four from the outlet of the syringe (Fig. 1). The samples were suspended with methanol and ultra-sonicated for 15 min. Two hundred microliters of the dissolved sample was mixed with 100 μl internal standard solution (amphetamine-d5, 100 ng/ml) and 40 μl 1 M NaOH solution. Subsequently, liquid–liquid extraction was conducted with 500 μl n-hexane and subsequent vortexing for 1 min. Phase separation was achieved by centrifugation at 8050 × *g* for 8 min, so that 160 μl of the organic phase could be transferred into a micro-vial. Derivatization was performed by the addition of 40 μl MBFTA to the organic phase and incubation of the sample for 30 min at 70 °C. Afterwards, the extract was stored for minimum 30 min at – 20 °C to achieve phase separation. Subsequently, the resulting MBFTA phase was injected into the gaschromatographic-mass spectrometric (GC–MS) system.

### Instrumentation

Qualitative substance analyses were performed by means of LC–MS/MS. The LC–MS/MS apparatus consisted of an LC-20 series HPLC system (binary pump, degasser, column oven, and autosampler; Shimadzu, Duisburg, Germany) coupled to an API 4000 QTrap mass spectrometer (Sciex, Darmstadt, Germany). An Allure® pentafluorophenyl propyl 5 μm (50 × 2.1 mm) LC-column (Restek, Bad Homburg, Germany) with a C18 ODS Octadecyl (4 mm × 2 mm) pre-column (Phenomenex, Aschaffenburg, Germany) was used. Separation was performed using gradient solution of solvent A (ammonium formate (1 M)/formic acid/deionized water (2:2:996, v/v/v)) and solvent B (ammonium formate (1 M)/formic acid/acetonitrile (2:2:996, v/v/v)). Ionization was performed by electrospray ionization (ESI). The LC–MS/MS system was operated in enhanced product ion (EPI) mode under three different collision energies (CE = 20, 35, and 50 eV). The assessment of detected spectra is performed by the comparison with a spectra database. The used spectra database is an in-house database, which covers the detection of approximately 300 forensic relevant substances.

Quantitative measurements for amphetamine were conducted on a GC–MS 5973 system (Agilent Technologies, Waldbronn, Germany) equipped with a splitless inlet and an autosampler PAL Combi-xt (CTC Analytics, Zwingen, Switzerland). A 4-mm ID TAP GW glass liner (Agilent Technologies, Waldbronn, Germany) and a GC column 19091S-433 HP 5 MS 15 m × 0.25 mm × 0.25 μm (both from Agilent Technologies, Waldbronn, Germany) were used. Separation was performed with a constant helium flow of 1.0 ml/min and the following temperature program: After an initial holding of the temperature of 120 °C for 0.5 min, the temperature was increased to 180 °C by constant heating of 15 °C/min, followed by the heating to 220 °C with 10 °C/min and further 90 °C/min to a final temperature of 300 °C, which was then held for 3 min. The GC–MS system was operated in single-ion monitoring (SIM) mode with electron ionization (70 eV). Detected single ions for amphetamine/amphetamine-d5 were m/z 140/144 (target), m/z 118/123 (qualifier 1), and m/z 91/92 (qualifier 2).

## Results

Amphetamine was not detected qualitatively in the first sample obtained from the inside of the syringe at the plunger seal. With regard to the positive result for amphetamine during the police-induced NIK®substance test, it was assumed that the police officers performed the test by pressing some parts of the substance out of the syringe. Therefore, the outlet of the syringe was used as an additional sampling site, leading to, positive findings for amphetamine. To exclude possible contaminations of the ointment, multiple samples were obtained from the different sampling sites on the inside of the syringe at the plunger seal as well as from the outlet. Amphetamine could be detected and quantified in all four samples received from the outlet of the syringe, but only in two out of three from the inside at the plunger seal.

The quantitative measurements were performed with the GC–MS method described above. The results are displayed in Table 1. Samples from the outlet of the syringe yielded median substance concentrations of approx. 15.3 ng/mg, whereby the fourth sample only contained approx. 7.2 ng/mg amphetamine. For the samples from the inside of the syringe at the plunger seal, amphetamine could only be detected in 2 of 3 samples at significantly lower concentrations (0.5 ng/mg and 0.8 ng/mg) compared to the concentrations detected at the syringe tip.

**Table 1:** Substance concentrations and corresponding weight percentage of amphetamine dependent on sampling site, used sample weight, and corresponding sample number

Sampling site	Sample number	Sample weight (mg)	Substance concentration (amphetamine) (ng/mg)	Weight percentage (% w/w)
Syringe outlet	1	38.0	Approx. 15.3	≈ 0.0015
	2	20.0	Approx. 17.4	≈ 0.0017
	3	33.7	Approx. 15.3	≈ 0.0015
	4	26.7	Approx. 7.2	≈ 0.0007
Syringe inside at the plunger seal	1	20.0	Not detected	-
	2	21.2	Approx. 0.5	< 0.0001
	3	24.4	Approx. 0.8	< 0.0001

With regard to the statement of the accused that the secured substance was cortisone cream, it has to be mentioned that glucocorticoids, including cortisone, would not have been detected with the performed analyses in the laboratory. Nevertheless, further (controlled) substances could not be detected in qualitative analyses via LC–MS/MS.

## Discussion

When comparing the amphetamine concentrations from the different sampling sites, significantly different results were obtained. Considering the guidelines of the GTFCh [4], a single exhibit sample is sufficient for qualitative substance determination, while at least 30% of the complete exhibit amount has to be homogenized and sampled for quantitative analyses. With regard to the presented case, negative substance findings in the first sample (from the inside of the syringe at the plunger seal) in association with the positive substance test performed by the police led to investigations on the different sampling sites to display possible contaminations of the exhibit. Hence, the measurements revealed up to 34-fold higher amphetamine concentrations at the outlet of the syringe in comparison to the samples obtained from the inside at the plunger seal.

If only qualitative analyses of a single sample (first sample) would have been performed in the present case, negative findings for amphetamine would be obtained. Accordingly, the results emphasize the importance of homogenization of controlled substance samples for accurate concentration determination on the one hand and also the importance of different sampling sites when contamination is worth considering. In relation to the latter, homogenization of the sample could have also been disadvantageous to the analyses and positive substance findings, respectively, since it might lead to reduced concentration. Hence, homogenization might have caused a concentration of amphetamine in the whole sample that would have been too low for detection.

Nevertheless, it has to be mentioned that the measured substance concentrations are relatively low compared to averaged substance concentrations of amphetamine in seizures of narcotics. Referring to the European Drug Report of the EMCDDA [1] from 2020 the averagely found amphetamine concentrations in narcotics in Europe were located between 13 and 67% in 2019, whereby one-half of the countries reported an average purity of 20–35%. In a publication by Losacker et al. [6], 79 seizures of amphetamine samples, mostly powders, were investigated. Median concentrations of amphetamine were 16.2%, in a range of 0.4 to 73.1%. With respect to the presented case, amphetamine concentrations in secured narcotics in Europe and in the work from Losacker et al. [6] were significantly higher compared to the drug concentrations found herein. Hence, the measured concentrations would rather comply with contamination of the syringe, especially on the outlet. It is conceivable that the ointment was either applied to a skin location where previous amphetamine skin contact occurred or that the syringe was deposited on a location where amphetamine had been consumed.

The fact that low amphetamine concentrations could also be detected on the inside of the syringe at the plunge seal might be explained by contaminations during the filling of the syringe. Another possible explanation might be an unintended mixing of the exhibit during the sampling procedure. Nevertheless, the measured concentrations and concentration differences in the sample most likely indicate contamination of the paste-like substance with amphetamine.

## Conclusion

This case report emphasizes the importance of the implication of different sampling sites in the analyses of (controlled) substance samples and narcotics, respectively, in forensic-toxicological issues. As described by the guidelines of the GTFCh, a homogenization of the exhibit is inevitable for correct analyte quantification. Nevertheless, the consideration of different sampling sites can be beneficial for, e.g., plausibility checks concerning possible contamination of the sample. In the presented case, measurements of samples from different sampling sites and the variable data indicated that positive amphetamine results have most likely aroused from contamination of the sample.
